# Recent advances and new perspectives in mitochondrial dysfunction

**DOI:** 10.1038/s41598-023-34624-8

**Published:** 2023-05-17

**Authors:** Cecilia Giulivi, Kezhong Zhang, Hirofumi Arakawa

**Affiliations:** 1 School of Veterinary Medicine, University of California at Davis and The MIND Institute, University of California Davis, Davis, CA USA; 2 Center for Molecular Medicine and Genetics, Wayne State University School of Medicine, Detroit, USA; 3 Division of Cancer Biology, National Cancer Center Research Institute, Tokyo, Japan

**Keywords:** Biochemistry, Biomarkers

## Abstract

In the last decade, there has been an increased appreciation for mitochondria as central hubs in diverse processes, such as cellular energy, immunity, and signal transduction. As such, we have become aware that mitochondrial dysfunction underlies many diseases, including primary (mutations in genes encoding mitochondrial proteins) and secondary mitochondrial diseases (mutations in non-mitochondrial genes critical for mitochondrial biology), as well as complex diseases with mitochondrial dysfunction (chronic or degenerative diseases). Evidence suggests that mitochondrial dysfunction may often precede other pathological signs in these disorders, further modulated by genetics, environment, and lifestyle.

This Collection aimed to gather research works providing insights into the mechanisms of bioenergetic deficits and potential therapeutic options. Our understanding of the role of mitochondrial dysfunction (MD) in disease has rapidly expanded in recent years^1–5^, providing new avenues for diagnosis, treatment, and prevention. Therefore, it is critical to understand the role of mitochondrial dysfunction in certain diseases to comprehend the impact of mitochondrial disorders on developing neurological diseases and how mitochondrial medicine can treat chronic diseases.

Among the excellent studies in this Collection, we would like to highlight a few that address some of the gaps in knowledge mentioned earlier.

In the context of mechanisms of prenatal adverse outcomes and MD, several studies pointed to the associations between serum-free beta chorionic gonadotropin and adverse maternal, fetal, and pregnancy effects (i.e., spontaneous abortion, intrauterine growth restriction, and preterm birth). Kiyokoba et al.^6^ reported high human chorionic gonadotropin (hCG) expression in placentae from pregnancies associated with fetal growth restriction with and without pre-eclampsia. This over-expression could result from HIF-1ɑ stabilization sustained by high oxidative stress because of syncytiotrophoblasts' low mitochondrial antioxidant capacity during the first eight weeks of pregnancy. The high levels of hCG result in deficits in mtDNA translation, leading to decreased bioenergetic capacity^6^, possibly contributing to fetal growth restriction. While this study addresses the mechanism of MD in pregnancies with relatively high hCG, more research must address the detrimental effect of abnormally low and high hCG levels, as both are generally associated with an increased risk of adverse pregnancy outcomes^7,8^.

Regarding treatments targeting mitochondrial dysfunction, three studies explored therapies to improve mitochondrial function. Two focused on Barth syndrome (BTHS), a congenital disease associated with early onset cardio-skeletal myopathy caused by mutations in the TAZ gene encoding the cardiolipin remodeling enzyme. Surprisingly, both studies provided new evidence to support that cardiolipin remodeling may not be required to improve mitochondrial function in this syndrome. One study^9^ showed that *N-*oleoyl-ethanolamide treatment significantly improved mitochondrial morphology and function of BTHS lymphoblasts, possibly by affecting mitochondrial dynamics without resolving cardiolipin alterations. The other one^10^ reported that SS-31 peptide improved mitochondrial respiratory capacity and promoted supercomplex organization in a BTHS animal model without affecting altered cardiolipin remodeling. Both studies showed a future therapeutic strategy that does not necessarily involve correcting cardiolipin remodeling deficits in BTHS.

Another study reported the importance of early mitochondrial metabolic defects and a therapeutic strategy to correct them in amyotrophic lateral sclerosis (ALS). TDP-43 is a versatile RNA/DNA binding protein involved in RNA-related metabolism. Hyper-phosphorylated and ubiquitinated TDP-43 deposits act as inclusion bodies in the brain and spinal cord of patients with motor neuron diseases, including amyotrophic lateral sclerosis (ALS) and frontotemporal lobar degeneration. Treatments for ALS are challenging because of the complexity of the disorder, which involves numerous mechanisms linked with progressive motor neuron degeneration, including glutamate-mediated excitotoxicity, protein aggregation, increased oxidative stress, endoplasmic reticulum stress, MD, neuroinflammation, and gene expression dysregulation^11^. Thus, targeting TDP-43 toxicity seems essential for finding therapies for these diseases when inclusion bodies are evident^11^. In this context, Gautam et al.^12^ reported that MD occurs early in ALS, as in other neurodegenerative diseases. Restoring NAD^+^ levels with nicotinamide mononucleotide improved the neurite length and mitochondrial cristae structure of ALS-affected murine corticospinal motor neurons. This therapeutical approach could become a new strategy that, if implemented early in ALS, may prevent the formation of inclusion bodies and the progression of the disease.

In pursuing effective therapies for neurodegenerative diseases, recent findings suggest that addressing metabolic dysfunction early in the disease may also hold promise for preventing overt symptoms or slowing the disease progression. Alongside these efforts, advances in AI technology offer exciting possibilities for enhancing disease diagnosis and treatment. Artificial intelligence (AI) techniques, such as convolutional neural networks, knowledge graphs, and transformers, can revolutionize our understanding and management of various medical conditions, including neurodegenerative diseases^13–16^. In this Collection, Giulivi et al.^17^ demonstrated the feasibility of an artificial neural network (ANN), based on mitochondrial bioenergetics and brain imaging, in predicting the progression of the neurodegenerative disorder fragile X-associated tremor/ataxia syndrome (FXTAS). Utilizing brain MRI data from patients and mitochondrial bioenergetic information from peripheral blood mononuclear cells and fibroblasts, ANN was able to make a suitable stage classification for FXTAS. If the ANN approach and outcomes are validated in larger cohorts, the predicted trajectories through combined analyses of cellular mitochondrial bioenergetics and brain imaging could represent a breakthrough in clinical screening and detection of FXTAS morbidity as well as other neurogenerative diseases.

In summary, the studies published in this Collection demonstrate how far our understanding of the pathogenic mechanisms, disease diagnosis, and management of affected individuals with mitochondrial disorders and conditions with mitochondria deficits has come in the last 5 years. However, these studies also highlight the work that needs to be done to improve our understanding and treatment of these disorders. Nevertheless, mitochondrial dysfunction represents a major mechanistic basis, predictive biomarker, and therapeutic target of various genetic, environmental, or age-associated complex diseases.
